# Lycopene Pretreatment Ameliorates Acute Ethanol Induced NAD^+^ Depletion in Human Astroglial Cells

**DOI:** 10.1155/2015/741612

**Published:** 2015-05-14

**Authors:** Jade Guest, Gilles J. Guillemin, Benjamin Heng, Ross Grant

**Affiliations:** ^1^Australasian Research Institute, Sydney Adventist Hospital, Sydney, NSW 2076, Australia; ^2^Department of Pharmacology, School of Medical Sciences, Faculty of Medicine, University of New South Wales, Sydney, NSW 2052, Australia; ^3^Neuropharmacology Group, MND and Neurodegenerative Diseases Research Centre, Macquarie University, Sydney, NSW 2109, Australia; ^4^Sydney Adventist Hospital Clinical School, University of Sydney, Sydney, NSW 2076, Australia

## Abstract

Excessive alcohol consumption is associated with reduced brain volume and cognition. While the mechanisms by which ethanol induces these deleterious effects *in vivo* are varied most are associated with increased inflammatory and oxidative processes. In order to further characterise the effect of acute ethanol exposure on oxidative damage and NAD^+^ levels in the brain, human U251 astroglioma cells were exposed to physiologically relevant doses of ethanol (11 mM, 22 mM, 65 mM, and 100 mM) for ≤ 30 minutes. Ethanol exposure resulted in a dose dependent increase in both ROS and poly(ADP-ribose) polymer production. Significant decreases in total NAD(H) and sirtuin 1 activity were also observed at concentrations ≥ 22 mM. Similar to U251 cells, exposure to ethanol (≥22 mM) decreased levels of NAD(H) in primary human astrocytes. NAD(H) depletion in primary astrocytes was prevented by pretreatment with 1 *μ*M of lycopene for 3.5 hours. Unexpectedly, in U251 cells lycopene treatment at concentrations ≥ 5 *μ*M resulted in significant reductions in [NAD(H)]. This study suggests that exposure of the brain to alcohol at commonly observed blood concentrations may cause transitory oxidative damage which may be at least partly ameliorated by lycopene.

## 1. Introduction

While the consequence of light-to-moderate alcohol consumption on brain health is currently debated within the literature, excessive consumption is generally agreed upon to reduce both brain volume and cognition [1, 2]. The ways in which ethanol may impose these deleterious effects are varied and although not completely understood most appear to be associated with an increase in inflammatory and oxidative processes. Within the brain this is thought to occur via several pathways including proinflammatory cytokine inducible nitric oxide synthase activation [3], decreased antioxidant enzyme activity [4], increased NMDA receptor sensitivity [5], cytochrome P450 2El induction [6], increased prostanoid production through cyclooxygenase/phospholipase A2 mechanisms [7, 8], and hydroxyethyl radical formation [9].

The shift in redox balance following ethanol exposure is known to cause a number of morphological and functional brain cell alterations including an increase in DNA nicks and breaks and subsequent activation of poly(ADP-ribose) polymerase (PARP) [10]. Importantly, in order to facilitate DNA repair, PARP uses nicotinamide adenine dinucleotide (NAD^+^) as a substrate to form poly(ADP-ribose) subunits. Under conditions of mild-to-moderate DNA damage this process promotes cell survival [11]. However overactivation of PARP, in response to extreme DNA damage, as may occur following alcohol consumption [12, 13], can cause both neuronal and astroglial death as a consequence of decreased ATP production due to NAD^+^ depletion [14, 15].

The importance of maintaining adequate concentrations of the ubiquitous NAD^+^ molecule for cellular health is the subject of increased interest. In addition to its role in generating cell energy (i.e., ATP) and metabolism there are several enzymes, in addition to PARP, which use NAD^+^ as their substrate. The sirtuins (SIRT), a family of protein deacetylases, are one such group that depend on NAD^+^ for their activity [16]. Known for their role in cellular senescence and aging, modulation of sirtuin activity via NAD^+^ dependent mechanisms has been shown to impact the course of several neurodegenerative diseases [17–19]. Sirtuins have also been identified as key inhibitors of microglia-mediated inflammation and oxidative damage [20]. Considered together, evidence suggests that, in order to maintain healthy brain cell activity through sufficient cellular energy production and appropriate PARP and SIRT activity, sufficient stores of NAD^+^ must be maintained.

Early investigations of the effect of ethanol on NAD^+^ metabolism have shown that excessive acute/chronic exposure can significantly decrease NAD^+^ stores in both* Drosophila* and rats [21, 22]. In line with this work we recently reported an inverse association between alcohol consumption (≥0-1 standard drink/day) and total nicotinamide adenine dinucleotide (NAD(H)) levels in the human CSF of free living adults [23]. Importantly in this same cohort, we also reported that increased levels of CSF [NAD(H)] were associated with high plasma concentrations of the diet derived phytochemical, lycopene [24].

Lycopene, a naturally occurring carotenoid found abundantly in red coloured fruits and vegetables, is a potent quencher of singlet oxygen (^1^O_2_) [25]. In hepatic HepG2 cells lycopene has been shown to attenuate alcohol-induced oxidative stress [26], in Mongolian gerbils, to protect the hippocampus against oxidative damage after ischemia-reperfusion [27]. Interestingly lycopene has also been demonstrated in murine models to restore PARP and SIRT activity following oxidative stimuli [28, 29]. Collectively, these findings suggest that lycopene may potentially act as a neuroprotective agent by attenuating reactive oxygen species (ROS) production and reducing oxidative damage, though these effects are yet to be demonstrated in human brain tissue.

In this study, we investigated the effect of acute ethanol exposure, at commonly observed blood alcohol concentrations (BAC), on oxidative processes and NAD(H) levels in cultured human brain glial cells. We demonstrated that at physiologically relevant levels lycopene is protective against acute ethanol toxicity by suppressing oxidative damage and subsequent PARP activity and thus preserving [NAD(H)].

## 2. Materials and Methods

### 2.1. Tissue Preparation and Cell Culture

Sixteen-to-nineteen week foetal brains were collected following therapeutic termination with informed consent. Mixed brain cultures were prepared and maintained according to ethical approval by HREC 08284, using a protocol previously described [30].

Astrocytes were prepared from the mixed brain cell cultures using a protocol previously described [31]. The human astroglioma U251 cell line was also used. Cells were cultured in RPMI 1640 media (Sigma-Aldrich) supplemented with 20% foetal bovine serum (Gibco), 1% L-glutamine (Gibco), and 1% antibiotic-antimycotic (Invitrogen) at 37°C in a humidified incubator containing 5% CO_2_ and 95% air. Cells were seeded into 96-well culture plates at a density of 2 × 10^4^ (astrocytes; P1–5) or 3 × 10^4^ (U251; P1–20) cells/well at least 12 hours prior to experimentation.

### 2.2. Cell Treatment

Cells were exposed to physiologically relevant concentrations of ethanol (11 mM, BAC 0.05%; 22 mM, BAC 0.1%; 65 mM, BAC 0.3%; and 100 mM, BAC 0.5%) for either 10, 15, or 30 minutes at 37°C. All treatments were administered as solutions in RPMI 1640 phenol red-free media, adjusted to a pH 7-8, and prepared immediately before dosing.

Lycopene (Sigma-Aldrich) was dissolved in tetrahydrofuran (Sigma-Aldrich) containing 0.025% butylated hydroxytoluene (Sigma-Aldrich) to inhibit formation of peroxides. This stock solution was prepared with minimal exposure to air and light and stored at −80°C. Immediately before the experiments, the lycopene stock solution was diluted in RPMI 1640 phenol red-free media (Sigma-Aldrich), homogenised, and filtered at 0.45 *μ*m. For lycopene dosage experiments cells were exposed to physiologically relevant concentrations of lycopene (0.1 *μ*M, 1.0 *μ*M, 5 *μ*M, and 10 *μ*M) for 3.5 hours at 37°C. To investigate the influence of lycopene on ethanol mediated effects, cells were exposed to 1.0 *μ*M of lycopene for 3.5 hours at 37°C. This dosage was chosen based on previously published data from our group indicating that normal physiological concentrations of lycopene within the central nervous system are < 1.5 *μ*M [24]. In addition high concentrations of lycopene have been shown to induce toxicity, particularly under prooxidant conditions, such as those produced during ethanol treatment [32, 33]. The amount of tetrahydrofuran/butylated hydroxytoluene vehicle in the culture medium was never greater than 0.1% (v/v), a concentration that has previously been shown not to affect cell number, viability, or ROS production [34].

### 2.3. Reactive Oxygen Species

Intracellular ROS were quantified using the cell-permeable fluorogenic probe 2′,7′-dichlorodihydrofluorescein diacetate (DCFH-DA) according to a method adapted from the OxiSelect Intracellular ROS Assay Kit (Green Fluorescence) (Cell Biolabs Inc.). Briefly, cells were cultured in a 96-well cell plate and then preincubated with DCFH-DA for 1 hour. The DCFH-DA diffuses into the cells where it is deacetylated by cellular esterases to nonfluorescent 2′,7′-dichlorodihydrofluorescein. Cultures were then exposed to the appropriate treatment after which cells were lysed using a 1% Triton-X lysis buffer. The fluorescence intensity was then measured at excitation/emission = 485/528 nm.

In the presence of ROS 2′,7′-dichlorodihydrofluorescein is rapidly oxidized to highly fluorescent 2′,7′-dichlorodihydrofluorescein. The resulting fluorescent signal is therefore proportional to the level of reactive oxygen species generated within the cell. Results are expressed as the change in absorbance relative to control.

### 2.4. Poly(ADP-Ribose) Polymers

Poly(ADP-ribose) polymers were quantified as a measure of PARP activity using the In-Cell ELISA Detection Kit (Thermo Scientific), according to the manufacturer's instructions. Briefly to quantify poly(ADP-ribose) polymers, cells were fixed after the appropriate treatment(s) using 4% formaldehyde (Sigma-Aldrich) and incubated at 4°C overnight in the presence of diluted (1 : 1000) anti-PADPR [10H] antibody (Abcam). Following the addition of a horseradish peroxidase conjugate and 3,3′,5,5′-tetramethylbenzidine substrate the absorbance was measured at 450 nm. The number of cells per well was then determined using the Janus Green Whole-Cell Stain. Results are expressed as the change in absorbance relative to control and adjusted for variations in cell number.

### 2.5. Sirtuin 1 Activity

As the experiments conducted in this study were acute (i.e., ≤ 30 minutes) and therefore unlikely to alter either protein or gene expression levels, only SIRT1 activity was quantified using the SensoLyte Green SIRT1 Assay Kit: Fluorimetric (AnaSpec) according to the manufacturer's instructions. In this protocol, the deacetylation of substrate by SIRT1 within the cell homogenate results in the generation of a fluorophore that was detected after the addition of a developer solution at *E*_*x*_/*E*_*m*_ = 490 nm/520 nm. Results are expressed as the change in absorbance relative to control and adjusted for variations in protein content.

### 2.6. NAD(H)

Intracellular NAD(H) levels were measured using the thiazolyl blue microcycling assay established by Bernofsky and Swan [35] and adapted for a 96-well plate format by Grant and Kapoor [36]. In this assay NAD^+^ present within the cell homogenate is converted to NADH, which in turn reduces thiazolyl blue tetrazolium bromide to a purple formazan product, which absorbs at 570 nm. The reaction mixture that causes this conversion consisted of phenazine methosulfate (2 mM; Sigma-Aldrich), thiazolyl blue tetrazolium bromide (0.5 mM; Sigma-Aldrich), ethanol (0.6 M), bicine (120 mM, pH 7.8; Sigma-Aldrich), and alcohol dehydrogenase (1 mg/mL; Sigma-Aldrich). Results are expressed as the change in absorbance relative to control and adjusted for variations in protein content.

### 2.7. Total Protein

Results for SIRT1 activity and NAD(H) assays are adjusted for variations in protein content, quantified using the Bradford protein assay. In this protocol Coomassie Brilliant Blue G-250 (Bradford reagent; Sigma-Aldrich) binds to protein causing a shift in the absorption maximum of the dye from 465 nm to 595 nm which was measured spectrophotometrically.

### 2.8. Statistical Analysis

Statistical analyses were performed using SPSS version 16.0 and GraphPad Prism version 5 for Windows. Data is presented as the mean ± SEM. Significant differences between treatments were assessed using one-way analysis of variance with post hoc Tukey Multiple Comparisons Test after verification of normality within the data set. If the data was not normally distributed the nonparametric Kruskal-Wallis with post hoc Dunn's Multiple Comparisons Test was employed. *P* values are provided throughout with test significance set at *P* value ≤ 0.05.

## 3. Results

### 3.1. Ethanol Increases the Production of ROS in U251 Cells

**Figure 1:**
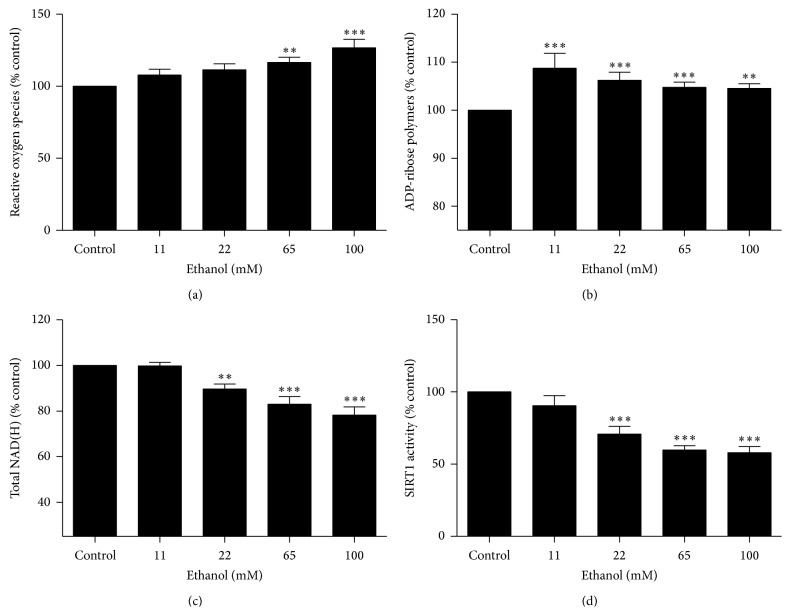
Ethanol exposure of U251 cells for ≤ 30 minutes, effects on (a) reactive oxygen species production, (b) generation of poly(ADP-ribose) polymers, (c) NAD(H) concentration, and (d) SIRT1 activity. ^∗∗^ *P* < 0.01, ^∗∗∗^ *P* < 0.001. Data are presented as mean ± SEM.

Exposure to 11 mM, 22 mM, 65 mM (*P* < 0.01), and 100 mM (*P* < 0.001) of ethanol for 10 minutes increased intracellular ROS production above control by 7.9 ± 3.9%, 11.5 ± 4.1%, 16.6 ± 3.6%, and 26.7 ± 5.8%, respectively; however statistically significant differences were only observed following exposure to 65 mM and 100 mM of ethanol (Figure 1(a)).

### 3.2. Ethanol Increases the Production of Poly(ADP-Ribose) Polymers in U251 Cells

Exposure to 11 mM (*P* < 0.001), 22 mM (*P* < 0.001), 65 mM (*P* < 0.001), and 100 mM (*P* < 0.01) of ethanol for 15 minutes increased poly(ADP-ribose) polymer production above control by 8.7 ± 3.1%, 6.2 ± 1.7%, 4.8 ± 1.1%, and 4.5 ± 1.0%, respectively (Figure 1(b)).

### 3.3. Ethanol Decreases Total NAD(H) in U251 Cells

Exposure to ethanol for 30 minutes was found to decrease the concentration of intracellular NAD(H) in the U251 cell line. Exposure to 22 mM (*P* < 0.01), 65 mM (*P* < 0.001), and 100 mM (*P* < 0.001) of ethanol decreased intracellular NAD(H) levels compared to control by 10.4 ± 2.2%, 17.0 ± 3.3%, and 21.8 ± 3.6%, respectively (Figure 1(c)).

### 3.4. Ethanol Decreases SIRT1 Activity in U251 Cells

Exposure to ethanol for 30 minutes was found to decrease SIRT1 activity in the U251 cell line. Exposure to 22 mM (*P* < 0.001), 65 mM (*P* < 0.001), and 100 mM (*P* < 0.001) of ethanol for 30 minutes decreased SIRT1 activity by 29.2 ± 5.4%, 40.2 ± 3.1%, and 42.0 ± 4.2%, respectively, compared to control (Figure 1(d)).

### 3.5. Lycopene Decreases Total NAD(H) in U251 Cells

**Figure 2:**
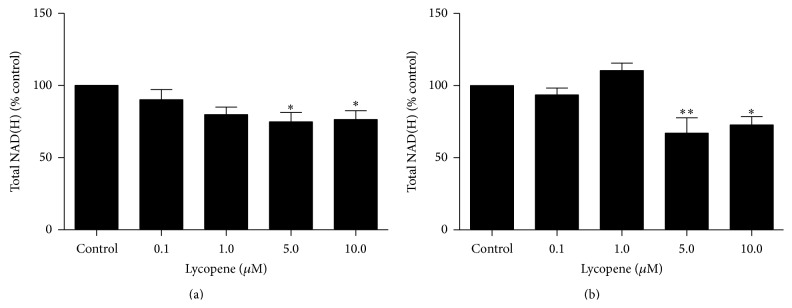
The effect of lycopene on NAD(H) concentrations in (a) U251 cells and (b) primary human astrocytes after 3.5 hours. ^∗^ *P* < 0.05, ^∗∗^ *P* < 0.01. Data are presented as mean ± SEM.

Exposure of U251 cells to 0.1 *μ*M, 1 *μ*M, 5 *μ*M (*P* < 0.05), and 10 *μ*M (*P* < 0.05) of lycopene for 3.5 hours resulted in a decrease in NAD(H) levels by 9.8 ± 7.0%, 20.2 ± 5.2%, 25.2 ± 6.5%, and 23.6 ± 6.2%, respectively (Figure 2(a)). However statistically significant reductions in NAD(H) were only observed following exposure to ≥ 5 *μ*M of lycopene.

### 3.6. Effect of Lycopene on Total NAD(H) in Primary Astrocytes

Unlike U251 cells, exposure to 0.1 *μ*M of lycopene for 3.5 hours did not affect NAD(H) levels in primary astrocytes. Although an apparent 10.4 ± 5.2% increase in [NAD(H)], compared to control, was observed after exposure to 1.0 *μ*M of lycopene, this was not statistically significant. Exposure to 5 *μ*M (*P* < 0.01) and 10 *μ*M (*P* < 0.05) of lycopene was found to reduce [NAD(H)] by 32.9 ± 10.7% and 27.2 ± 5.8%, respectively, compared to control (Figure 2(b)).

### 3.7. Ethanol Decreases Total NAD(H) in Primary Astrocytes

**Figure 3:**
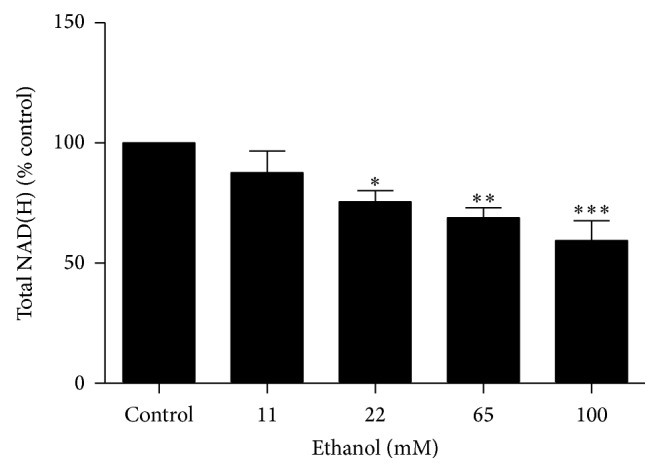
The effect of ethanol on NAD(H) concentrations in primary human astrocytes after 30 minutes. ^∗^ *P* < 0.05, ^∗∗^ *P* < 0.01, ^∗∗∗^ *P* < 0.001. Data are presented as mean ± SEM.

Similar to U251 cells, exposure to ethanol for 30 minutes decreased the concentration of intracellular NAD(H) in primary astrocytes (Figure 3). Exposure to 22 mM (*P* < 0.05), 65 mM (*P* < 0.01), and 100 mM (*P* < 0.001) of ethanol decreased NAD(H) levels, compared to control, by 24.4 ± 4.6%, 31.0 ± 4.0%, and 40.6 ± 8.2%, respectively.

### 3.8. Lycopene Ameliorates Ethanol Induced NAD(H) Depletion in Primary Astrocytes

**Figure 4:**
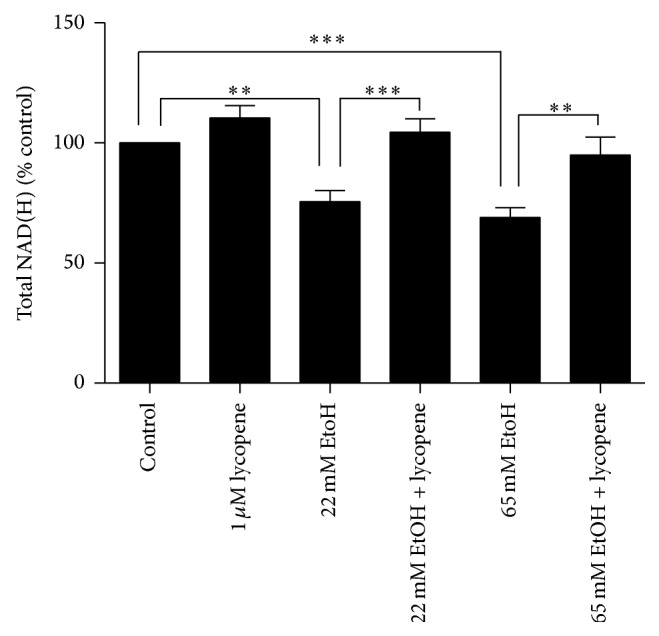
Pretreatment of primary human astrocytes with lycopene (1 *μ*M) for 3.5 hours preserved the ethanol (22 mM, 65 mM) facilitated decrease in NAD(H) concentrations. ^∗^ *P* < 0.05, ^∗∗^ *P* < 0.01, and ^∗∗∗^ *P* < 0.001. Data are presented as mean ± SEM.

In order to determine if lycopene could prevent the ethanol induced depletion in [NAD(H)], primary astrocytes were pretreated with 1 *μ*M of lycopene for 3.5 hours. Pretreated cells were then exposed to either 22 mM or 65 mM concentrations of ethanol for 30 minutes. Pretreated primary astrocytes exposed to either 22 mM (*P* < 0.001) or 65 mM (*P* < 0.01) concentrations of ethanol maintained intracellular NAD(H) levels at 104.4 ± 5.7% and 95.0 ± 7.4%, respectively (Figure 4).

## 4. Discussion

There is strong evidence to suggest that excessive acute and/or chronic alcohol intake results in damage to the brain. Nevertheless the mechanisms underlying ethanol's deleterious effects are not completely understood. In this study we investigated the effect of acute ethanol exposure, at physiologically relevant concentrations, on oxidative stress and NAD(H)-related biochemistry in human astrocytes and sought to determine if any ethanol induced reductions in [NAD(H)] could be ameliorated by lycopene. We found that acute ethanol exposure dose dependently increased the production of ROS in U251 cells. This result is in line with several previous* in vitro* and* in vivo* studies where both acute and chronic ethanol exposure were found to increase oxidative activity in neurons and astrocytes as well as various cerebral regions [37–39]. However, while the majority of previous studies were conducted using high ethanol doses, the results from the present study suggest that ethanol may, at least temporarily, enhance oxidative activity within exposed brain cells at physiologically relevant concentrations.

Ethanol exposure was also observed to cause a significant increase in U251 cell PARP activity. This effect was observed at concentrations as low as 11 mM, a dose equivalent to a blood alcohol concentration of 0.05%, attained by consumption of approximately two standard drinks. Although few reports detail ethanol's ability to induce astroglial DNA damage in the form that activates PARP, Cherian and colleagues previously observed that ethanol treatment of neurons generates an increase in DNA nicks which was associated with a marked upregulation of PARP expression [10]. Consistent with these findings a significant increase in poly(ADP-ribose) polymers was apparent in the present study after only 15 minutes of exposure to 11 mM of EtOH reflecting the initiation of DNA repair in response to ethanol induced oxidative damage and DNA strand breaks.

As previously discussed, in order to facilitate DNA repair, PARP uses NAD^+^ as its key substrate. Accordingly, we observed a dose dependent relationship between increasing ethanol concentrations and decreased [NAD(H)]. While research investigating the effect of ethanol on NAD(H) is scarce these results are consistent with previous reports by us showing reduced levels of [NAD(H)] in CSF of free living individuals who consume ≥ 0-1 standard drink of alcohol per day [23]. Early investigations in which NAD^+^ levels were observed to decrease in response to ethanol stress further corroborate the findings from the present study [21, 22].

The sirtuins proteins are a family of enzymes that, like PARP, use NAD^+^ as their primary substrate. While ethanol exposure has previously been shown to reduce SIRT1 activity in the murine liver [40, 41] and is thus hypothesised to be an important target of ethanol action, surprisingly no known study has previously investigated the effect of ethanol on SIRT activity in the brain or its cells. In this study acute ethanol exposure was found to reduce SIRT1 activity in U251 cells in a dose dependent manner. This novel effect is consistent with the observed PARP facilitated NAD^+^ depletion. While a decrease in SIRT1 activity may also occur as a result of an ethanol induced reduction in SIRT protein synthesis [42], this is unlikely to have occurred in this study due to the short (≤ 30 minutes) duration of ethanol exposure. Further research is required to identify the precise mechanism involved.

Considered together these findings indicate that acute ethanol exposure (≤ 30 minutes), at physiologically relevant concentrations, increases ROS production in U251 cells leading to an increase in PARP activity, presumably in response to DNA damage, and a subsequent decrease in NAD(H) and SIRT activity.

We then sought to determine if the ethanol induced shift in redox balance and consequential decrease in NAD(H) stores could be ameliorated by known diet derived antioxidants like lycopene, a phytonutrient. Unexpectedly, exposure of U251 cells to lycopene resulted in a dose dependent decrease in [NAD(H)], suggesting a reduction in cell viability. Though this affect is not known to have been previously observed in these astroglioma U251 cells, this data is consistent with reports by others showing that lycopene can induce cell cycle arrest and increase apoptosis in cancer cell lines of the breast, colon, and prostate [43]. While the finding from the present study supports the proposal that lycopene may be protective against various cancers, further investigation is required to determine its therapeutic potential and was beyond the scope of this study. To more effectively investigate whether lycopene could be protective against ethanol induced damage in brain cells, we again tested this hypothesis in cultures of primary human astrocytes.

In contrast to the detrimental effect of lycopene on U251 cells, exposure of primary astrocytes to commonly observed blood lycopene concentrations (≤ 1 *μ*M) [24] was not found to alter NAD(H) levels. Exposure to supraphysiological concentrations (≥ 5 *μ*M) did however result in a reduction in [NAD(H)]. This is consistent with results from an investigation by Qu and colleagues, where primary neuron viability was not observed to significantly decrease until a lycopene dosage of 10 *μ*M was applied, although nonsignificant reductions were observed following exposure to 5 *μ*M [32]. Similarly, albeit in a human colon cancer cell line (HT29), Lowe found that lycopene only afforded protection against DNA damage (induced by xanthine/xanthine oxidase) at relatively low concentrations (1–3 *μ*M). At higher concentrations (4–10 *μ*M), the ability to protect cells against such oxidative damage was rapidly lost, with the presence of lycopene instead appearing to increase the extent of DNA damage [33].

A number of mechanisms have been suggested as reasons for lycopene's toxicity at high concentrations. In conditions of enhanced oxidative potential lycopene has been shown in human fibroblasts to act as a prooxidant, increasing lipid peroxidation at dosages ≥ 10 *μ*M [44]. Unlike low dose lycopene supplementation (1.1 mg/kg), high dose supplementation (3.3 mg/kg) has also been shown to significantly induce hepatic CYP2E1 protein (a known producer of ROS and an ethanol metaboliser), TNF-*α* mRNA, and the incidence of inflammatory foci in alcohol fed rats [45]. While further* in vivo* research is required, these studies indicate that at supraphysiological concentrations lycopene may potentiate toxicity and suggest a need for caution when supplementing, particularly among individuals with enhanced oxidative potential.

Despite some evidence suggesting potential toxicity of lycopene at high doses, the antioxidant properties of lycopene are well documented [25–27] and have been extensively reviewed by others [46–49]. Indeed in the present study pretreatment of primary astrocytes with 1 *μ*M of lycopene was found to preserve intracellular [NAD(H)] following acute ethanol exposure. This is consistent with a previous report by Xu et al. [26] who demonstrated that lycopene can prevent ethanol induced oxidative stress in hepatic cells, as well as* in vivo* data by our laboratory of a positive association between human plasma lycopene concentrations (0.4 ± 0.3 *μ*M) and CSF [NAD(H)] [24]. Considering the evidence we speculate that this novel finding is most likely the consequence of lycopene's potent ROS quenching ability.

Out of the 600 known carotenoids, lycopene is considered one of the most efficient singlet oxygen quenchers [25]. Lycopene is also known to effectively scavenge hydroxyl radicals, capable of abstracting a hydrogen atom from ethanol resulting in the production of 1-hydroxyethyl, as well as superoxide anions and hydroperoxides, both of which are produced during ethanol metabolism and are, at least partially, responsible for ethanol's adverse effects [50, 51]. In addition to directly interacting with ROS, lycopene has been shown to inhibit oxidative damage by modulating ROS generating enzymes (such as nicotinamide adenine dinucleotide phosphate-oxidase, inducible nitric oxide synthase, cytochrome P450 enzymes, and cyclooxygenase-2) and by activating protective/antioxidant Phase II enzymes (including heme oxygenase-1, NAD(P)H dehydrogenase quinone 1, and glutathione S-transferase) [52–56].

## 5. Conclusions

In summary, results from this study indicate that acute ethanol exposure, at physiologically relevant concentrations, increases oxidative damage in U251 cells, resulting in upregulation of PARP activity and consequently a significant decrease in intracellular levels of its substrate NAD(H), a molecule critical for cellular health. A novel dose dependent relationship between ethanol exposure and reduced concentrations of the protective NAD^+^ dependent enzyme SIRT1 was also observed. Further investigation is required to determine if this effect was solely the consequence of reduced NAD^+^ availability. While data from this study suggests that exposure of the brain to alcohol at commonly observed blood concentrations may cause at least transitory oxidative damage, the novel finding that lycopene can prevent the ethanol induced reduction of astrocyte NAD(H) stores suggests that this naturally occurring carotenoid, if present in the brain at around 1 *μ*M, may at least partly ameliorate ethanol mediated brain toxicity. Further research using mixed culture and murine models is required to confirm this hypothesis.

## Abbreviations

BAC: Blood alcohol concentration
DCFH-DA: 2′,7′-Dichlorodihydrofluorescein diacetate
NAD^+^: Nicotinamide adenine dinucleotide
NAD(H): Total nicotinamide adenine dinucleotide
PARP: Poly(ADP-ribose) polymerase
ROS: Reactive oxygen species
SIRT: Sirtuins.
